# Evaluation of anxiolytic activity of compound *Valeriana jatamansi* Jones in mice

**DOI:** 10.1186/1472-6882-12-223

**Published:** 2012-11-21

**Authors:** Jie-Shu You, Min Peng, Jin-Li Shi, Hu-Zhan Zheng, Yong Liu, Bao-Sheng Zhao, Jian-You Guo

**Affiliations:** 1 School of Chinese Materia Medica, Beijing University of Chinese Medicine, Beijing 100102, P.R. China; 2 Key Laboratory of Mental Health, Institute of Psychology, Chinese Academy of Sciences, Beijing 100101, P.R. China

**Keywords:** Compound *Valeriana jatamansi* Jones, Anxiolytic, Elevated maze-plus, Light–dark box, Benzodiazepine receptors

## Abstract

**Background:**

Compound *Valeriana jatamansi* Jones is a formula for treating anxiety-related diseases in the clinic, which is composed of *Valeriana jatamansi* Rhizoma et Radix, *Ziziphi Spinosae* Semen, *Albiziae* Cortex and Junci Medulla. The purpose of this study was to explore the anxiolytic properties of this compound in mice.

**Methods:**

Male ICR mice were treated with compound *Valerianae Jatamansi* Jones (1.2 g/kg, 2.4 g/kg, 4.8 g/kg), saline, diazepam (2 mg/kg) orally for 10 days and then exposed to elevated maze-plus (EPM) and light–dark box (LDB). The effects of the compound on spontaneous activity were evaluated by locomotor activity test. We further investigated the mechanism of action underlying the anxiolytic-like effect of compound by pre-treating animals with antagonists of benzodiazepine (flumazenil, 3mg/kg) prior to evaluation using EPM and LDB.

**Results:**

Compound *Valerianae Jatamansi* Jones (2.4, 4.8 g/kg, p.o.) significantly increased entries (*P*<0.05) into and time spent (*P*<0.05) on the open arms of the EPM, and number of transitions (*P*<0.05) and time spent (*P*<0.05) in the light compartment of the LDB. However, the anxiolytic-like effects of compound were significantly reduced by pre-treatment with flumazenil (*P*>0.05). In addition, compound *Valerianae Jatamansi* Jones treatment didn’t affect the spontaneous activity in mice (*P*> 0.05).

**Conclusions:**

The present study supports the hypothesis that compound *Valeriana jatamansi* Jones exert anxiolytic action but no sedative effects in mice and that this effect might be mediated by benzodiazepine receptors.

## Background

Anxiety disorders are the most common and prevalent behavioral disorders that can result in significant impairment of function and quality of life, and they affect 17.2% of the population in the United States [1]. Currently, pharmacotherapy is the most widespread and efficacious treatment for anxiety disorders [2]. Benzodiazepines are the most common class of compounds used for anxiety [3]. However, the short-term use of benzodiazepines interferes with the formation of new memories. Moreover, it can induce complete anterograde amnesia [4]. Studies reported that between 20–100% of patients experienced withdrawal symptoms, sedation, and dependence when they took benzodiazepines at therapeutic doses for prolonged periods [5]. Therefore, the development of other anxiolytic drugs without the adverse effects of benzodiazepines would significantly improve the treatment of anxiety disorders.

Traditional Chinese Medicine has been commonly recognized as a safe and effective means of treating anxiety disorders [6-8]. The compound V*aleriana jatamansi* Jones is used to treat anxiety-related diseases [9], and is composed of *Valeriana jatamansi* Rhizoma et Radix (12g), *Ziziphi Spinosae* Semen (9g), *Albiziae* Cortex (9g) and Junci Medulla (1g). The anxiolytic effects of this formulation have been previously established in the rat [10]. However, considering its low affinity for benzodiazepine binding site in binding assays, it is unclear whether this anxiolytic activity was mediated by this site modulation at γ-aminobutyric acid (GABA)-A receptors. In addition, chromatographic profiling of this formulation for purity was not carried out in previous study [10].

Therefore, in this study, we further explored the anxiolytic effects of compound *Valeriana jatamansi* Jones in mice using the elevated plus maze (EPM), light/dark box (LDB) test, and spontaneous activity. The formulation was first evaluated for purity by high-performance liquid chromatography (HPLC). We also examined whether its anxiolytic effects are mediated by GABA(A) receptors through co-administration of the benzodiazepine antagonist flumazenil.

## Materials and methods

### Plant material

*Valeriana jatamansi* Rhizoma et Radix was purchased from a commercial source in Yunnan province, China. *Ziziphi Spinosae* Semen, *Albiziae* Cortex, and *Junci* Medulla were purchased from a commercial source in Hebei province, China. The identity of the herbal medicine was confirmed by Professor Shi, a researcher in the Department of Pharmacognosy, Beijing University of Chinese Medicine. Voucher specimens were deposited at the Herbarium of School of Chinese Materia Medica, Beijing University of Chinese Medicine.

### Preparation of the extracts

Previous chemical and pharmacological studies in our laboratory demonstrated that the compound *Valeriana jatamansi* Jones has optimum efficacy when the four herbal components were extracted by different solvents. *Jatamana Valeriana Rhizome* was homogenized to coarse powder, refluxed three times each for 2h in aqueous ethanol (35%, 2L, v/v), and the combined alcoholic extract was filtered and evaporated (yield 20.7% (w/w)). *Albiziae* Cortex and *Ziziphi Spinosae* Semen were also homogenized to coarse powder, infused in 2L of cold distilled water, and extracted for 3h twice by heating. Then, the extracts were concentrated to dryness. The yield of the extracts of *Albiziae* Cortex and *Ziziphi Spinosae* Semen were 15.2% and 25.8% (w/w), respectively. *Junci* Medulla was refluxed for 1.5h in aqueous ethanol (95%, 1.5L, v/v) three times, and the combined alcoholic extract was filtered and concentrated (yield 4.5% (w/w)). Based on the original formulation and the herb extraction yield, the four extracts (*Valeriana jatamansi* Rhizoma et Radix, *Ziziphi Spinosae* Semen, *Albiziae* Cortex and Junci Medulla) were mixed with a ratio of 5:3:5:1 to get the compound Valeriana jatamansi Jones.

### Chromatographic conditions of HPLC

9.80 mg hesperidin reference substance (Lot 110721–200512, the National Institute for the Control of Pharmaceutical and Biological Products, China) was transferred into a 25 mL measuring flask and mixed with methanol. Then, the mixture was shaken and filtered through 0.45μm filters. 5g of mixed dry extracts were used to prepare the sample solution and refluxed in an appropriate amount of methanol to a colorless extract and filtered. Then, it was transferred to a 100mL measuring flask and brought up to level with methanol and filtered through 0.45μm filters. 5μL solutions were injected. The samples were chromatographed using an Agilent® C18 chromatographic column (5 μm, 250 × 4.6mm), a mobile phase consisting: acetonitrile - 0.3% phosphoric acid (20∶80, v/v), a flow rate of 1mL/min, a column temperature of 35°C, and UV detection at 280nm.

### Drugs

The following drugs were used in the study: diazepam hydrochloride (2.5mg/tab.), three different concentrations (1.2, 2.4, 4.8 g/kg) of the compound *Valeriana jatamansi* Jones and flumazenil (Sigma, St Louis, MO, USA). For oral (p.o.) administration for 10 days, compound Valeriana jatamansi Jones and diazepam were dissolved in saline. Each drug was administered in a volume of 0.4mL/25g body weight. Control animals received vehicle (saline, 0.4mL/25g) only. For evaluating the anxiolytic effects of the compound *Valeriana jatamansi* Jones, the mice were administered the compound *Valeriana jatamansi* Jones (1.2, 2.4, 4.8 g/kg) or diazepam 60 and 30min before the test, respectively. For exploring the anxiolytic mechanisms of the compound *Valeriana jatamansi*, the mice were also administered the compound *Valeriana jatamansi* Jones (1.2, 2.4, 4.8 g/kg) or diazepam for 10 days described above. On the last day, flumazenil (GABA(A) antagonist) was co-administered (i.p., 3mg/kg) 30 min before the test. All of the animal tests were performed on the 10th day of treatment.

### Animals

Male Imprinting Control Region (ICR) mice weighing 18–22g were purchased from the China Academy of Military Medical Sciences and kept in cages of 5 mice at 22 ± 1°C on a 12-h light/dark cycle (with lights on from 08:00–20:00). Water and food were available *ad libitum*. Groups of 12–20 mice were randomly assigned to different treatment groups and tested in a counterbalanced order. All experiments were carried out in a quiet room under a dim red light between 8:00 and 14:00. The experimental procedures were approved by the Institutional Animal Care and Use committee of the Institute of Psychology of the Chinese Academy of Sciences and in accordance with the National Institutes of Health Guide for Care and Use of Laboratory Animals. All efforts were made to minimize the number of animals used and their suffering.

### Elevated plus-maze

Anxiolytic activity was measured using the elevated plus-maze procedure [11]. The maze consisted of two opposite open (30 cm×5 cm×0.2 cm) and two opposite closed (30 cm×5 cm×15 cm) arms, extending from a central platform (5 cm ×5 cm) and elevated to a height of 45 cm above the floor. The entire maze was made of clear Plexiglas. Mice were individually placed on the center of the maze facing an open arm, and the number of entries and the time spent in the closed and open arms were recorded during a 5 min observation period. Arm entries were defined as the placement of all four paws into an arm. The percentage of open-arm entries (open/total entries×100) was calculated for each animal. The compound *Valeriana jatamansi* Jones and saline were administered orally 1 h before testing and the positive controls were treated with diazepam 30 min before evaluation in the maze. All tests were carried out on the tenth or last day of treatment. After each trial, the apparatus was cleaned with 30% alcohol.

### Light/dark box

The apparatus (45×21×21cm) consisted of two compartments with one third painted white and two thirds painted black, and these compartments were separated by a divider with a 3.5×3.5cm opening at floor level [12,13]. The white compartment was illuminated by 2×60 W lamps. Each mouse was gently placed in the corner of the white area away from the dark chambers and monitored over a 5-min observation period. The number of transfers from one compartment to the other and the time spent in the illuminated side was measured. All sessions were recorded by a camera linked to a monitor in an adjacent room to avoid distractions. After testing each mouse, the apparatus was thoroughly cleaned with both wet and dry cloths.

### Spontaneous activity

To rule out any unspecific locomotor effects of the compound *Valeriana jatamansi* Jones on our measures of anxiolytic effects, the activity of individual mouse was recorded by a computer-based system (Anilab, Ningbo, China) for locomotor activity.

### Statistical analysis

The data are expressed as the mean ± S.E.M. The statistical analysis was carried out by a one-way analysis of variance (ANOVA) followed by Student-Newman-Keul’s post-hoc tests using Prism 5.0 (Graphpad Software, Inc). Significance was accepted at a P <0.05.

## Results

### HPLC assays

**Figure 1:**
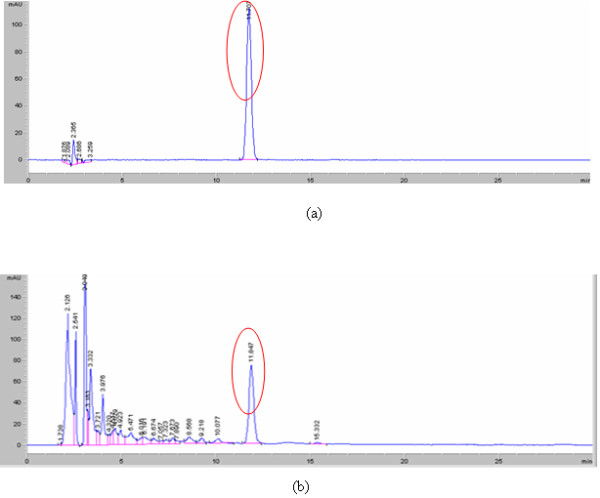
HPLC profile of Hesperidin(a) and sample solution(b) using acetonitrile - 0.3% phosphoric acid (20∶80, v/v) at 1.0mL/min on a Agilent ® C18 column, 5 μm, 150 × 4.6mm , 35°Cwith UV detection at 280 nm.

As *Valeriana jatamansi* Jones was used as monarch drug, hesperidin content was selected for the quality control components. The results showed that the hesperidin in the sample produced a well-resolved peak, and the resolution with its adjacent chromatographic peaks was more than 1.5 (Figure 1). For linearity, stock solutions of 1μL, 2μL, 3μL, 4μL, 5μL, and 8μL were injected. The calibration curve was prepared with the absolute amount (μL) as independent variable (X) and the peak area of Hesperidin as dependent variable (Y). The corresponding regression equations was computed and found to be: Y=1625.5X +45.087, R=0.9999. A linear relationship was obtained in the range of 0.392–3.136 μg. To test the precision of the assay, reproducibility was calculated by analyzing 6 samples. The precision results showed a RSD of 0.44% (n=6). To test the stability of the assay, the sample solution was analyzed every 2 h over 10 h (n =6). RSD=0.72% (n=6).

### Elevated plus-maze

**Figure 2:**
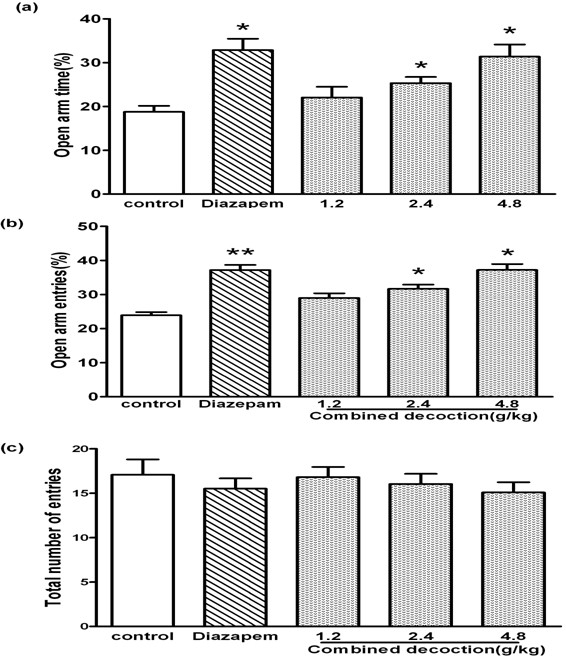
**Behavioral outcome of compound *Valerianae Jatamansi *Jone during 5 min on the elevated plus-maze.** (**a**) The percentage of time spent in open arms. (**b**) The percentage of open arms entries. (**c**) The total number of entries. Bars represent mean ± S.E.M. *P<0.05 or **P<0.01 vs. control group. One way ANOVA with Student-Newman-Keul’s post hoc test.

**Figure 3:**
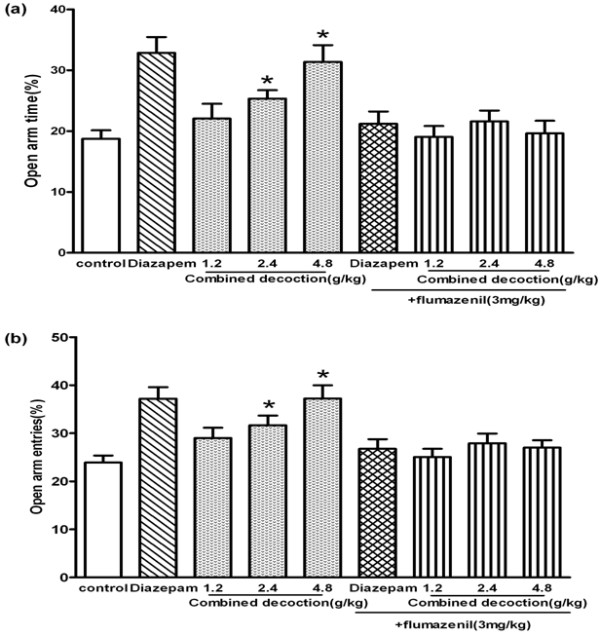
**Behavioral outcome of flumazenil on the anxiolytic-like effect of compound *Valerianae Jatamansi *Joneduring 5 min on the elevated plus-maze.** (**a**) The percentage of time spent in open arms. (**b**) The percentage of open arms entries. Bars represent mean ± S.E.M. *P<0.05 or **P<0.01 vs. control group. One way ANOVA with Student-Newman-Keul’s post hoc test.

A one-way ANOVA indicated significant differences among the groups in the time spent in the open arms of the elevated plus maze (*F*_4,72_=7.45, *P*<0.01; Figure 2a) and the percentage of open-arm entries (*F*_*4,72*_*=*16.70, *P* < 0.01; Figure 2b). Compound *Valeriana jatamansi* Jones at concentrations of 2.4 and 4.8 g/kg significantly increased the percentage of time spent in the open arms and the number of entries into the open arm, compared to the saline-treated group (*P* < 0.05). No differences were observed in the total arm entries among all groups (*F*_*4,72*_*=*0.89, *P*>0.05; Figure 2c). After the mice treated with compound *Valeriana jatamansi* Jones were co-administered flumazenil, there were no differences in the percentage of time spent in the open arms (*F*_4,72_=0.49, *P*>0.05; Figure 3a) or the number of entries into the open arm (*F*_4,72_=0.84, *P*>0.05; Figure 3b) among all groups.

### Light–dark box test

**Figure 4:**
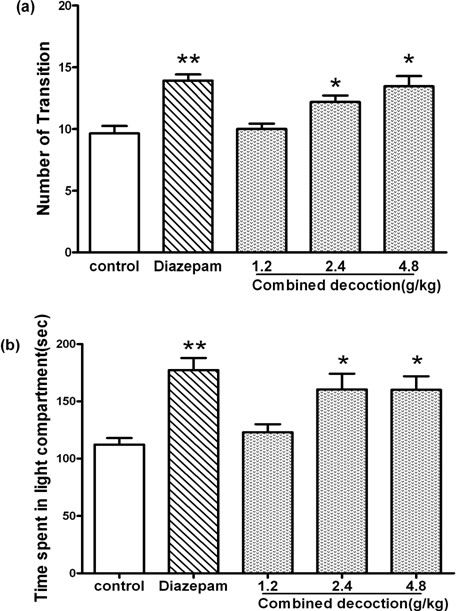
**Behavioral outcome of compound *Valerianae Jatamansi *Jone during 5 min in the light–dark box.** (**a**) The numbers of transition. (**b**) Time spent in light compartment. Bars represents mean ± S.E.M. *P<0.05 or **P<0.01 vs. control group. One way ANOVA with Student-Newman-Keul’s post hoc test.

**Figure 5:**
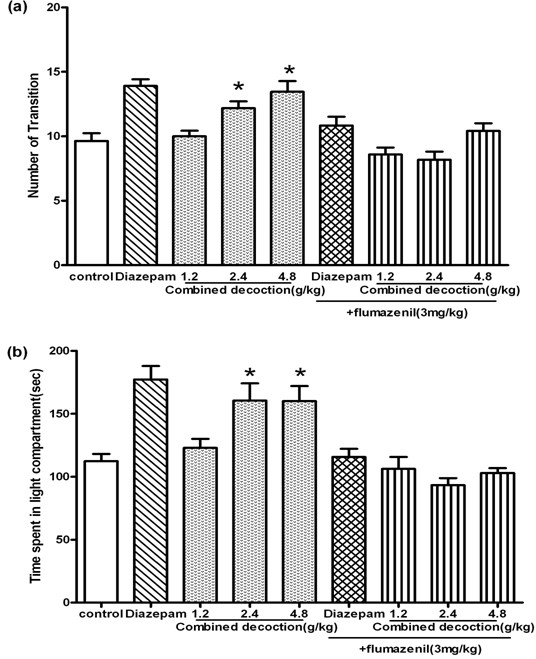
**Behavioral outcome of flumazenil on the anxiolytic-like effect of compound *Valerianae Jatamansi *Jone during 5 min in the light–dark box.** (**a**) The numbers of transition. (**b**) Time spent in light compartment. Bars represents mean ± S.E.M. *P < 0.05 vs. control group. One way ANOVA with Student-Newman-Keul’s post hoc test.

As shown in Figure 4, one-way ANOVA revealed significant differences among the five groups in both the time spent in the light compartment (*F*_4,69_=7.236, *P* < 0.01) and the number of transitions between compartments (*F*_4,69_=10.73, *P*< 0.01). Compared with the saline-treated mice, treatment with compound *Valeriana jatamansi* Jones at doses of 2.4g/kg and 4.8g/kg significantly increased the time spent in the light compartment and the number of transitions between compartments (both *P*< 0.05). After the mice treated with compound *Valeriana jatamansi* Jones were co-administered flumazenil (3mg/kg; i.p.), the time spent in the light compartment (*F*_4,69_=1.805, *P*>0.05; Figure 5a) and the number of transitions between compartments (*F*_4,69_=1.531, *P*>0.05; Figure 5b) was reduced.

### Spontaneous activity

**Figure 6:**
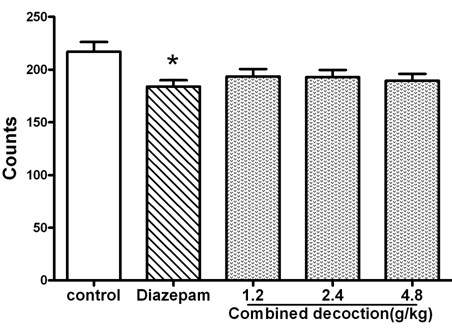
**The counts of compound *Valerianae Jatamansi *Jone during 5 min in spontaneous activity.** Bars represents mean ± S.E.M. of activity counts in 5 min *P < 0.05 vs. control group. One way ANOVA with Student-Newman-Keul’s post hoc test.

A one-way ANOVA indicated significant differences among the groups in the time spent in the open arms of the elevated plus maze (*F*_4,72_=3.123, *P*<0.05; Figure 6). Diazepam significantly reduced spontaneous activity in animals at 6 mg/kg (*P*<0.05) compared to the saline group. However, there were no significant differences among saline-treated animals and those treated with compound *Valeriana jatamansi* Jones at 1.2, 2.4, or 4.8 g/kg (*P*>0.05).

## Discussion

Based on a previous study that showed anxiolytic effects of compound *Valeriana jatamansi* Jones in rats [10], we further tested the anxiolytic effects of this formulation in mice using the EPM and LDB. We also further investigated the mechanism of action underlying the anxiolytic-like effect of compound by pre-treating animals with antagonists of benzodiazepine (flumazenil, 3mg/kg) prior to evaluation using EPM and LDB. We found that compound *Valeriana jatamansi* Jones exhibited anxiolytic-like activity, which may be related to the modulation of GABA(A) receptors. The combination of our results and those of a previous study strongly support the idea that compound *Valeriana jatamans*i Jones produces anxiolytic effects without inducing sedative side effects. These findings may support the development of potent and selective anxiolytic agents.

The EPM is one of the most popular animal models of anxiety [14,15]. It has been widely used to screen drug effects [16-18] because of its use of natural conditions and stimuli to induce anxiety, such as a fear of a new, bright, and open space and the fear of balancing on a relatively narrow raised surface [19]. This study also used the EPM model to evaluate the anxiolytic effects of compound *Valeriana jatamansi* Jones. As expected, compound *Valeriana jatamansi* Jones (2.4 and 4.8 g/kg) significantly increased time spent and entries into the open arms of EPM. Moreover, the anxiolytic effects of the compound *Valeriana jatamansi* Jones (4.8 g/kg) were similar to those of diazepam (2mg/kg). The main potential confounding factors in this procedure are changes in basal locomotor activity, which can be inferred from the total number of entries. However, the compound *Valeriana jatamansi* Jones did not alter total arm exploration in the elevated plus maze, suggesting that this drug induces specific anxiolytic-like effects.

The light/dark box test is another widely used animal model of anxiety. It is based on the innate aversion of rodents to bright areas and on the spontaneous exploratory behaviors of rodents in response to novel environments and light [13]. Previous studies reported that benzodiazepines can increase the transitions between the two compartments and the time spent in the light compartment, indicating anxiolytic effects [20,21]. In the present study, anxiolytic activity of the combined extract was observed at doses of 2.4 and 4.8g/kg in mice. Both of these doses significantly increased the number of transitions between the compartments and the time spent in the light compartment of LDB.

GABA (A) receptors are the most prevalent inhibitory neurotransmitter receptors in the brain. Most of them have separate modulatory sites that are sensitive to benzodiazepines. Benzodiazepines receptor agonists are commonly prescribed for the treatment of insomnia, anxiety disorders, and seizures, and these effects can be blocked by the antagonist flumazenil. In a previous binding assay [10], the binding of [3H]Ro 15–1788 (flumazenil) to the benzodiazepine binding site in washed crude synaptosomal membranes from rat cerebral cortex was affected by compound *Valeriana jatamansi* Jones. These data indicate that the anxiolytic-like effects of compound *Valeriana jatamansi* Jones may be mediated by benzodiazepine binding site modulation at GABA(A) receptors. However, the EC_50_ of this formulation is approximately 0.5 mg/mL. As such, it is hard to say whether this is a sufficient potency to produce *in vivo* neurochemical and behavioral actions, as the repeated oral application of compound by g/kg doses may not result in sufficient brain concentrations. In this study, we intraperitoneally administered flumazenil to mice 30 min before testing [22-24]. We observed that the anxiolytic-like effects of compound were significantly reduced by pre-treatment with flumazenil. These results further support the hypothesis that the anxiolytic effects of compound *Valeriana jatamansi* Jones are mainly mediated via benzodiazepine receptors.

Valerian is the main ingredient of this formulation, and it is one of the most popular herbal supplements for the treatment of anxiety and insomnia in Europe [25]. The sedative effects of this formulation and the positive control were tested in the present study. However, we found that the dose of 2 mg/kg of diazepam did not produce sedative effects in our preliminary experiments. To compare the sedative effects between a positive control drug and compound *Valeriana jatamansi* Jones, the dose of diazepam was increased to 6 mg/kg in the locomotor activity test. Although this dose of diazepam significantly reduced spontaneous activity counts, none of the three doses of compound *Valeriana jatamansi* Jones affected spontaneous activity in the locomotor test. As such, we found that compound *Valeriana jatamansi* Jones induced anxiolytic effects with an absence of sedative effects. This may be because the doses tested were too low to yield sedative effects. In addition, McKernan *et al*. showed that the GABA(A) receptor α_1_ subtype mediates the sedative, but not the anxiolytic effects of benzodiazepines [26]. According to this finding, compound *Valeriana jatamansi* Jones may be more selective than diazepam, and not act through the α_1_ subtype but via other GABA_A_ receptor subtypes.

It should be noted that the three doses tested (1.2, 2.4 and 4.8 g/kg) are related to the original formulation but not to the extract. In the clinic, the dose of the compound *Valeriana jatamansi* Jones that is used is 31g per day. When this human dose is converted into an animal dose (a person of 60 kg, and a conversion factor of 9.01 between human and mice), it was equivalent to 4.8 g/kg. Therefore, 4.8 g/kg was chosen as the highest dose tested in this study, which is equal to 934 mg/kg extract of compound *Valeriana jatamansi* Jones**.** Unlike the regular formulation extraction process, *Valeriana jatamansi Rhizoma* et *Radix*, *ziziphi spinosae Semen*, *Albiziae Cortex,* and *Junci Medulla* were extracted by different solvents and methods. This was because the active constituents of these drugs are all sensitive to water temperature. An orthogonal experimental design method was performed to optimize the extraction process of the four herbs as described previously [27]. Briefly, in an L_9_ (3^4^) orthogonal test, the solid–liquid ratio, ethanol concentration, solvent amount, extraction times were determined. The extraction process of *Valeriana jatamansi* Rhizoma et Radix or *Junci* Medulla was determined by the content of hesperidin and the total extract. The optimum extraction condition of *ziziphi spinosae* Semen or *Albiziae* Cortex was determined by the content of jujuboside A. According to the original formulation and the herb extracting yield, the four extracts (*Valeriana jatamansi* Rhizoma et Radix, *Ziziphi Spinosae* Semen, *Albiziae* Cortex and Junci Medulla) were then mixed with a ratio of 5:3:5:1 to get the compound *Valeriana jatamansi* Jones.

As a positive control drug, diazepam exerted anxiolytic effects after a single dose (2 mg/kg, p.o.). However, in the present study, the anxiolytic activity was examined after the mice were given compound *Valeriana jatamansi* Jones for 10 days. Since previous studies have shown that many drugs elicited anxiolytic effects after administration for 7 days [28,29], it is possible to test the anxiolytic activity of the formulation using the EPM and LDB after administration for less than 10 days. In addition, previous researchers have indicated that etiology of anxiety may be related to the levels of monoamine neurotransmitters and neuroendocrine system [30]. Therefore, further studies are needed to identify these anxiolytic mechanisms.

## Conclusions

The present study further supports the hypothesis that compound *Valeriana jatamansi* Jones exert anxiolytic action but no sedative effects in mice and that this effect might be mediated by benzodiazepine receptors.

## Abbreviations

EPM: Elevated plus-maze; LDB: Light–dark box test; GABA: γ-aminobutyric acid; HPLC: High-performance liquid chromatography.

## Competing interests

The authors declare that they have no competing interests.

## Authors’ contributions

JSY and MP participated in the pharmacodynamic and mechanic studies drafted the manuscript. HZ, YL and BSZ participated in the design of the study and performed the statistical analysis. JLS and JYG conceived of the study, and participated in its design and coordination and helped to draft the manuscript. All authors read and approved the final manuscript.

## Pre-publication history

The pre-publication history for this paper can be accessed here:

http://www.biomedcentral.com/1472-6882/12/223/prepub
